# Diversity climate enhances work outcomes through trust and openness in workgroup communication

**DOI:** 10.1186/s40064-016-2499-4

**Published:** 2016-06-14

**Authors:** Joep Hofhuis, Pernill G. A. van der Rijt, Martijn Vlug

**Affiliations:** Department of Corporate Communication, University of Amsterdam, Nieuwe Achtergracht 166, 1018 WV Amsterdam, The Netherlands

**Keywords:** Workplace diversity, Diversity climate, Diversity outcomes, Workgroup communication, Trust, Openness, Knowledge sharing

## Abstract

Diversity climate, defined as an organizational climate characterized by openness towards and appreciation of individual differences, has been shown to enhance outcomes in culturally diverse teams. To date, it remains unclear which processes are responsible for these findings. This paper presents two quantitative studies (*n* = 91; 246) that identify trust and openness in workgroup communication as possible mediators. We replicate earlier findings that perceived diversity climate positively relates to job satisfaction, sense of inclusion, work group identification and knowledge sharing in teams. In study 1, trust is shown to mediate the effects of perceived diversity climate on team members’ sense of inclusion. In study 2, trust mediates the relationship between perceived diversity climate and workgroup identification and openness mediates its relationship with knowledge sharing.

## Introduction

During the past decades, the management of cultural diversity has emerged as one of the most prominent challenges facing modern organizations. A large number of studies have been conducted to determine the effects of diversity on organizational effectiveness, but findings have been ambiguous (see Van Knippenberg and Schippers 2007; Williams and O’Reilly 1998 for an overview). On the one hand, the presence of individual differences between employees appears to have the potential to enhance flexibility, creativity and innovation in workgroups (De Dreu and West 2001; Van Knippenberg et al. 2004). However, in many studies, diverse teams are reported to be outperformed by homogenous teams, because cultural differences may lead to lower cohesion, less effective interpersonal communication and a higher occurrence of conflict between team-members (Fiske 1998; Hofhuis et al. 2014; Williams and O’Reilly 1998).

Scholars have been in search of factors which may reduce diversity-related problems in the workplace, while still being able to take advantage of the potential benefits. One of the most promising constructs that has emerged from the literature is diversity climate, which can be defined as an organizational climate characterized by openness towards and/or appreciation of individual differences. This type of climate has been shown to positively affect outcomes on the individual, group, and organizational level (Buttner et al. 2012; Hofhuis et al. 2012; Mckay et al. 2007; Nakui et al. 2011). However, the processes through which diversity climate enhances outcomes remain largely unknown (Boehm et al. 2014; Dwertmann et al. 2016). In this paper, we will examine whether communication between members of diverse teams may be responsible for some of these findings. Two aspects of workgroup communication are identified which may play an important role in this regard: trust and openness. In two quantitative studies, we will test whether these constructs mediate the relationship between perceived diversity climate and job satisfaction, inclusion, workgroup identification and knowledge sharing.

## Theoretical background

### Outcomes of diversity in workgroups

As mentioned, existing literature reveals that diversity can simultaneously have both positive and negative effects on workgroup functioning (Van Knippenberg and Schippers 2007; Williams and O’Reilly 1998). The positive outcomes of diversity are often explained through the information-elaboration paradigm (Van Knippenberg et al. 2004): diverse teams are able to draw from a larger pool of available knowledge and experience, which enhances workgroup effectiveness. Expression of diverse opinions may force team members to be more alert and critical in their evaluation of problem solving strategies (Brodbeck and Greitemeyer 2000; Collins and Geutzkow 1964). This, in turn, may result in a reduced risk of groupthink and more effective decision making (De Dreu and West 2001; Nijstad and De Dreu 2002; West 2002). As such, positive diversity outcomes include increased knowledge sharing, flexibility, creativity and, as a result, better team performance. Empirical studies have found evidence for this relationship, in both experimental and organizational settings (De Dreu and West 2001; Kurtzberg and Amabile 2000; Milliken et al. 2003; Nakui et al. 2011).

However, diversity does not automatically lead to information elaboration and increased performance. Research rooted in the social identity/self-categorization paradigm (*Self*-*Categorization Theory*, Turner 1985; *Social Identity Theory*, Tajfel and Turner 1986) reveals that diversity may in fact also lead to negative outcomes in work groups (Van Knippenberg et al. 2004). When individuals identify with a social in-group, this is usually done on the basis of shared characteristics. Individuals who appear different are categorized as belonging to an out-group. This categorization helps individuals to predict and give meaning to their social environment and a positive evaluation of one’s in-group as compared to out-groups can provide a source of self-esteem. The downside of social categorization is that it also leads to the emergence of stereotypes and group representations that tend to favor the in-group over the out-group (Fiske 1998). Studies have shown that in culturally diverse organizations, employees often display a relative preference towards members who belong to the same cultural group (Brewer and Brown 1998; Goldberg 2005), which has a negative impact on interpersonal communication between members of different cultures (Dinsbach et al. 2007) and reduces employees’ sense of inclusion and organizational identification (Jansen et al. 2014; Pless and Maak 2004). As such, categorization into cultural subgroups lies at the heart of many of the negative outcomes of diversity in organizations, including reduced social cohesion, lower organizational commitment, and an increased chance of miscommunication and conflict (Fiske 1998; Milliken and Martins 1996; Pelled et al. 1999).

To summarize, the literature shows that diversity in workgroups leads to positive outcomes in terms of flexibility and creativity—through elaboration—as well as negative outcomes in terms of less fluent social interactions and reduced team cohesion—through categorization (Van Knippenberg et al. 2004). The challenge for modern organizations, therefore, is to find a way to reduce negative outcomes, while still retaining the benefits of diversity. In this regard, one of the most promising constructs which has emerged from the recent literature is the organizational climate with regard to diversity (Groggins and Ryan 2013).

### Diversity climate

Traditionally, diversity climate has been defined in terms of the presence of organizational policies and structures that promote the position of designated groups, such as affirmative action and career advancement programs (Chrobot-Mason and Aramovich 2013; Dwertmann et al. 2016; Groggins and Ryan 2013). This type of diversity climate has been related to favorable business outcomes like employee retention, customer satisfaction and sales performance (McKay et al. 2008; McKay et al. 2011). Recently, the definition of diversity climate has shifted to include more subjective constructs, such as employee attitudes, beliefs, and perceptions towards cultural differences (Van Knippenberg et al. 2013). A recent literature review by Dwertmann et al. (2016) reveals increasing use of a ‘synergy perspective’, which incorporates the notion that diversity leads to higher performance into the very definition of diversity climate. In line with this movement, the present research employs a definition based two separate components. Firstly, a strong diversity climate is characterized by the possibility of employees to freely discuss their cultural heritage and display cultural behaviors in the workplace. Secondly, diversity climate encompasses the belief that cultural differences provide added value to the team or organization, and that diversity is actively promoted. Although these components can be measured separately, earlier studies which use this definition show that they correlate strongly and should be viewed as two aspects of one overarching construct of diversity climate (see also Hofhuis et al. 2012; Luijters et al. 2008). This type of diversity climate also appears to relate to favorable outcomes such as satisfaction, inclusion and performance (Boehm et al. 2014; Gonzalez and Denisi 2009; Hofhuis et al. 2012; Schachner et al. 2016).

It must be noted that the way diversity climate is operationalized varies strongly across studies. In some cases, scholars make use of an aggregated score of diversity climate perceptions in groups or organizations, which is in line with the common definition of an organizational climate (Schneider et al. 2013). However, in the majority of studies (see Dwertmann et al. 2016) diversity climate is operationalized on the individual level, by measuring respondents’ individual perceptions of diversity attitudes and practices in their organization (e.g. Hofhuis et al. 2012). In these cases, which also include the studies described in this paper, diversity climate should be seen as a psychological climate variable, and treated as such (cf. Martin et al. 2005; Parker et al. 2003). Therefore, in this paper, when using the term diversity climate, the authors are referring to a construct that could technically be termed the *psychological climate towards diversity* within the workgroup or organization.

Furthermore, the definition of diversity climate as used in the present research is related to some other types of climate measures commonly mentioned in diversity literature. For example, the diversity climate as defined in this study may be linked to *ethical climate* (Victor and Cullen 1988), which refers to perceived norms and values in workgroups, and the broader *moral climate*, as described by Macklin et al. (2014), which also encompasses perceptions/evaluations of just behavior in the workplace. Using the definition as presented above, it follows that diversity climate is strongest when the ethical climate of a workgroup or organization allows for a wider range of acceptable behavior (thus allowing for diverse cultural heritages to be displayed/expressed) as well as providing justice to all groups in the organization, regardless of their cultural background. This is in line with an earlier comparison of diversity climate to a *low*-*prescription climate* (Luijters et al. 2008; Cox 1993) which allows for more diverse behavior and a high tolerance for ambiguity, thereby reducing some of the categorization-related problems that occur in diverse groups.

### Diversity climate and workgroup outcomes

When examining which types of outcome variables have been linked to diversity climate as defined above, it becomes apparent that most studies have sprung from the social identity/self-categorization paradigm (Dwertmann et al. 2016). For example, a strong diversity climate is reported to enhance workgroup involvement (Hobman et al. 2004) and team identification (Luijters et al. 2008), and reduce interpersonal aggression, miscommunication and diversity-related conflict (Drach-Zahavy and Trogan 2013; Gonzalez and Denisi 2009; Hofhuis et al. 2012). The direct link between diversity climate and information elaboration has not been examined empirically, but some studies report a relationship of positive diversity attitudes with brainstorming success and favorable evaluation of different viewpoints (Hofhuis et al. 2016; Homan et al. 2008; Nakui et al. 2011).

Although the body of evidence for the positive effects of diversity climate is growing, only a handful of studies examine the processes through which these effects come to be. For example, Boehm et al. (2014) show that the relationship between diversity climate and workgroup performance is mediated by a reduction in workplace discrimination. Hofhuis et al. (2012) report similar findings, and provide evidence that a strong diversity climate increases the ability of minority members to identify with the organization, which in turn leads to better job outcomes. A similar rationale is provided by Gonzalez and DeNisi (2009) who argue that diversity climate reduces the impact of categorization into in-groups and out-groups, thus reducing conflict and miscommunication in diverse teams. Based on these results, scholars have suggested that interpersonal processes, and workgroup communication in particular, could be the mediators through which diversity climate enhances workgroup outcomes (Groggins and Ryan 2013; Hofhuis et al. 2012; Luijters et al. 2008; Van Knippenberg et al. 2013), but this premise has not been tested empirically.

### Diversity climate and workgroup communication

Studies on interpersonal communication in a diverse workplace are scarce. Examples include research by Dahlin et al. (2005), who find an association between team diversity and greater information use, and by Dinsbach et al. (2007) who report that in the workplace, cultural minority employees communicate less about personal topics, and more about work-related issues than majority employees. A more dynamic view is promoted by Orbe (1998), who argues that minority employees adjust their communication strategies to match their preferred relationship with the (dominant) majority group. Both Dinsbach et al. (2007) and Wanguri (1996) describe that interpersonal communication in diverse organizations is positively affected by perceived equality between groups.

Surprisingly, these processes have rarely been linked to climate variables. In some papers on diversity climate, workgroup communication is suggested as a possible underlying process leading to diversity outcomes (e.g. Hofhuis et al. 2012; Luijters et al. 2008) but not empirically measured as a separate variable. Conversely, communication variables such as openness in leadership communication (e.g. Kearney and Gebert 2009; Sadri and Tran 2002) have been mentioned in combination with positive diversity outcomes, but not with the construct of diversity climate. In sum, very little is known about how diversity climate relates to communication in diverse workgroups, and how this may in turn affect diversity outcomes. The present research aims to fill this knowledge gap.

Reviewing the literature on cultural diversity on the one hand, and on interpersonal communication on the other, two main characteristics of workgroup communication appear to play an important role in both streams of research: trust and openness.

### Trust

Trust has proven essential in team-building within any kind of team (Costa et al. 2001). It has been defined as ‘the willingness of a party to be vulnerable to the actions of another party’ (Mayer et al. 1995). With regard to interpersonal communication, trust has been framed mostly as an affective construct, for example in terms of the relative comfort and psychological safety when communicating with others (Edmondson 1999; Singh et al. 2013), or the ease of information exchange or ‘flow’ in interpersonal contact (Butler 1999; Van Oortmerssen et al. 2014). Trust appears to relate to honesty, integrity and benevolence in communication, and affirms positive relationships between communicating parties (Mayer et al. 1995).

In diversity literature, trust is often mentioned as an outcome variable related to cohesion and team identification in diverse teams. Van der Zee et al. (2009) review the literature on diversity and trust, and state that cultural differences between team members may result in lower trust, due to categorization processes and the psychological effects of dissimilarity (see also Hooghe et al. 2009). They also propose, however, that a group climate which promotes positive diversity attitudes may turn around this relationship. Indeed, diversity climate has been shown to increase psychological safety, a construct which is closely related to trust in communication (Singh et al. 2013). Based on these arguments, we propose that when the climate reflects a positive view on diversity, team members may report more trust when communicating with fellow team members.

#### **Hypothesis 1**

Diversity climate is positively related to Trust in workgroup communication.

### Openness

Openness in communication has been defined as an organizational norm which promotes free disclosure of information (Eisenberg and Witten 1987). It has been related to a ‘low-prescription’ environment in which ideas are freely discussed and evaluated without judgement (Luijters et al. 2008; Patterson et al. 2005). Openness also appears to encompass the possibility to use different communication styles and channels, depending on the situation, thereby increasing the degree of flexibility in interpersonal communication that is considered appropriate in the workplace (Rogers 1987).

In diversity literature, openness is often defined as the freedom to express one’s cultural heritage in the workplace (e.g. Cox 1993) or the possibility to openly discuss cultural differences between team members, as well as the problems that may arise from them (Luijters et al. 2008). It has been shown that diversity climate opens up the possibility to actively express different viewpoints and display culturally specific behaviors (Hofhuis et al. 2015), which in turn may increase flexibility in communication styles. Putting the two streams of research together, it is logical to assume that a strong diversity climate, in which cultural differences are encouraged and seen as valuable, is also related to more open communication between members of different cultural groups.

#### **Hypothesis 2**

Diversity climate is positively related to openness in workgroup communication.

It is important to note that trust and openness, while defined here as separate components of workgroup communication, are not independent constructs per se. A correlation between trust and openness has been reported by several scholars (Butler 1999; Frey and Luethje 2011; Greenhalgh and Chapman 1998), but discussion remains about the direction of a possible causal relationship. Ruppel and Harrington (2000) found support for their hypothesis that a greater level of trust in the organizational subunit leads to more open communication among managers and employees. Conversely, Tjosvold (1999) suggests that open-minded discussion leads to productive collaborative work, which in turn results in trust. Although a reciprocal relationship may exist, the present research views trust and openness as two related yet separate concepts, with trust seen as a more affective, and openness as a more behavioral construct.

Finally, both trust and openness in communication have been related to positive workgroup outcomes in terms of job satisfaction, inclusion and innovation (e.g. Smidts et al. 2001). Considering the existing evidence that diversity climate displays similar effects on outcomes, it is logical to assume that an increase in trust and openness may be (at least partly) responsible for these findings.

### Research overview

The abovementioned hypotheses were tested in two studies, which allowed for examining the relationships between diversity climate and workgroup communication in different contexts. Furthermore, the two studies examine the effects of diversity climate on different team outcomes. In Study 1, a survey was distributed among employees in small workgroups within a highly culturally diverse work environment. It examines the effects of diversity climate on job satisfaction and inclusion, and tests the mediating roles of openness and trust. To increase generalizability of our findings, Study 2 aimed to replicate the relationship between diversity climate and workgroup communication among a sample of employees working in a wide variety of different organizations and sectors. Furthermore, it tests the effects of diversity climate on workgroup identification and knowledge sharing, and providing further insight into the mediating roles of openness and trust.

## Study 1

### Introduction

The first aim of study 1 is to examine the relationship between diversity climate and trust and openness in workgroup communication. As explained above, we predict a positive relationship for both variables. Two outcome variables were included in this study: job satisfaction and inclusion.

It is a well-known finding in cross-cultural psychology that when individuals have the opportunity to display their cultural heritage, their satisfaction increases. In similar vein, studies have provided evidence for a positive relationship between diversity climate and job satisfaction, in a variety of settings (e.g. Choi 2013; Hofhuis et al. 2012; Madera et al. 2013). Hofhuis et al. (2012) show that this effect occurs for both majority and minority members, but is strongest for the latter group. Similar findings have been reported for inclusion: an environment which stimulates and appreciates the presence of individual differences allows individuals to feel a greater degree of belongingness, even when they perceive themselves as being different (Jansen et al. 2014; Roberson 2006).

In this study we hypothesize similar positive effects of diversity climate on these variables. Furthermore, existing studies have established that trust in workgroup communication has positive effects on both job satisfaction (De Vries et al. 2006) and inclusion of team members (Smidts et al. 2001). Similar results have been found for openness (Shore et al. 2011). As such, the second aim of study 1 is to test whether the positive effects of diversity climate on these outcome variables is mediated by trust and openness.

#### **Hypotheses 3–4**

Diversity climate is positively related to job satisfaction, mediated by trust (3) and openness (4) in workgroup communication.

#### **Hypotheses 5–6**

Diversity climate is positively related to Inclusion, mediated by trust (5) and openness (6) in workgroup communication.

### Methods

#### Procedure and sample

The hypotheses outlined above were tested in a survey study among a sample of employees working within production teams of a popular syndicated television show. A single location is used to simultaneously produce the show for different national audiences, forcing close cooperation between members of different cultural groups within multinational production teams. A total of 160 individuals worked at this location, divided into seven workgroups, each of which was highly culturally diverse. A research assistant worked within one of the teams, and distributed the survey on paper to 140 employees.

A total of 91 individuals fully completed the survey (response rate = 65 %). Of this sample, 68 % was male, mean age was 32.2 (SD = 8.7; range 20–53), and 52.7 % had received higher education, defined as having a bachelor’s degree or higher. The sample consisted of individuals from the Netherlands (48 %), Norway (22 %), Denmark (12 %), Belgium (9 %), Sweden (5 %), South Africa (2 %), the Philippines (1 %) and France (1 %).

#### Measures

All scales described below were measured using a Likert scale, ranging from 1 (*Strongly disagree*) to 5 (*Strongly agree*). The survey was written in English, which was the main working language in this setting.

*Diversity Climate* was measured using items which were adapted from a Dutch-language scale originally generated by Kruithof (2001), which was subsequently improved upon by Luijters et al. (2008) and Hofhuis et al. (2012). In this study, we used English translations (by the authors) of four of the items by Hofhuis et al. (2012). These items were formulated as follows: ‘In this organization there is room to work according to one’s own culture’, ‘In this organization we take into account different cultural traditions and habits of employees’, ‘In this organization it is seen as an advantage to work with people of different cultural backgrounds’ and ‘In this organization we appreciate different cultural backgrounds’. The scale was sufficiently reliable (*α* = .63).

*Trust* and *Openness* in workgroup communication were measured using scales from an instrument intended to measure components of an organizational communication climate (Bartels 2006). Only the subscales for Trust (four items, e.g. ‘Colleagues in my workgroup are honest to each other.’) and Openness (four items, e.g. ‘When I talk to colleagues in my workgroup, it feels safe to discuss anything’) were used. The scales were sufficiently reliable (*α* = .72; .73 respectively).

*Job Satisfaction* was measured using four items translated from De Witte (2000), including statements such as ‘I feel satisfied with my job at [organization]’. The scale was reliable (*α* = .81).

*Inclusion* was measured using three items by Jansen, et al. (2014), including statements such as ‘My workgroup gives me the feeling that I belong’. The scale was reliable (*α* = .84).

#### Measurement model

Because the constructs in this study are theoretically linked, we performed a confirmatory factor analysis to test the discriminant validity of the scales for diversity climate, trust, openness, job satisfaction and inclusion. We conducted these and all subsequent analyses using IBM SPSS Amos version 21.0 (Arbuckle 2012). Assessment of model fit was done using guidelines provided by Schermelleh-Engel et al. (2003).

First, we tested a one-factor model to assess whether respondents viewed the constructs as the same. This model produced a poor fit (*χ*^*2*^(142) = 286.282; *p* = .001; CFI = .758; RMSEA = .099). Next we tested a five-factor model using the intended constructs, which produced a good fit with the data (*χ*^*2*^(142) = 160.876; *p* = .133; CFI = .966; RMSEA = .038). Based on these findings, all five constructs are included in our model as intended. Table 1 provides descriptive statistics and intercorrelations of all variables in the model.

**Table 1:** Descriptive statistics and correlations of variables in study 1 (n = 91)

Variable	Descriptives			r				
	α	M	SD	(1)	(2)	(3)	(4)	(5)
Diversity climate (1)	.63	4.04	.86	–	.41***	.38***	.31**	.37***
Trust (2)	.71	3.89	.51		–	.24*	.45***	.57***
Openness (3)	.73	3.41	.53			–	.21*	.26**
Job satisfaction (4)	.81	3.95	.71				–	.64***
Inclusion (5)	.84	3.90	.69					–

* p < .05; ** p < .01; *** p < .001

### Results

#### Structural model

**Fig. 1:**
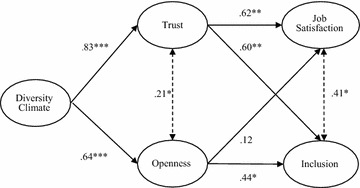
Structural model of effects of diversity climate on job satisfaction and inclusion, mediated by trust and openness (n = 91). *Note* A *double arrow* with a *dotted line* signifies a correlation between error terms of the latent variables; all reported coefficients are standardized; *χ* ^*2*^(144) = 163.495; *p* = .127; CFI = .965; RMSEA = .039; * p < .05; ** p < .01; *** p < .001

A structural equation model was constructed based on the hypotheses outlined above, including *Diversity Climate* as an independent variable, *Job Satisfaction* and *Inclusion* as dependent variables, and *Trust* and *Openness* as mediators. This model produced a good fit with the data (*χ*^*2*^(146) = 171.643; *p* = .072; CFI = .954; RMSEA = .044). However, modification indices showed that the model fit could be significantly improved by also including error correlations between *Trust* and *Openness,* as well as between *Job Satisfaction* and *Inclusion,* which implies these variables covary and may share a common cause which is not included in the model. This is not surprising, considering the theoretical overlap between the two variables, which was mentioned earlier. To accommodate for this overlap, a new model was constructed that included these error correlations (see Fig. 1), which provided a significant improvement over the hypothesized model (Δ*χ*^*2*^(2) = 8.148; *p* = .017).

##### Direct effects

Figure 1 shows the estimated standardized regression weights in the final model, which can be used to test our hypotheses. A strong positive relationship of diversity climate with both trust (b* = .83; SE = .29; *p* < .001) and openness (b* = .64; SE = .25; *p* < .001) was found, confirming both hypotheses 1 and 2. Furthermore, the results show that trust is positively related to both satisfaction (b* = .62; SE = .22; *p* < .005) and inclusion (b* = .60; SE = .20; *p* < .003). Openness displays a positive relationship with inclusion (b* = .44; SE = .19; *p* = .021), but not with job satisfaction (b* = .12; SE = .18; *p* = .231). Trust and openness display a positive correlation (*r* = .21; *p* = .045), as well as job satisfaction and inclusion (*r* = .41; *p* = .015).

##### Mediations

Hypotheses 3–6 predict that the positive effects of diversity climate on job satisfaction and inclusion are mediated by trust and openness. These hypotheses were tested using the phantom variable approach, as described by Macho and Ledermann (2011). By constructing latent variables for each mediating path, it is possible to calculate indirect effects of the independent variable on each dependent variable, through each mediator separately, using bootstrapping (5000 iterations).

Our results show a significant total effect of diversity climate on job satisfaction (b* = .54; *SE* = .11; *p* < .001). Hypotheses 3 and 4 predict that this effect is mediated by trust and openness. Our analyses reveal an indirect effect through trust (b* = .26; *SE* = .12; *p* = .060), but this is only significant at the α = .10 level. Hypothesis 3 is therefore rejected, but these finding do suggest it may be worthwhile to re-test this hypothesis using a larger sample. No indirect effect was found through openness, rejecting hypothesis 4.

Hypothesis 5 and 6 predict an indirect effect of diversity climate on inclusion, again mediated by trust and openness. Diversity climate displays a positive total effect on inclusion (b* = .69; *SE* = .09; *p* < .001). A significant indirect effect was found through trust only (b* = .29; *SE* = .11; *p* = .041), thus confirming hypothesis 5. The relationship between diversity climate and inclusion is not mediated by openness, thus hypothesis 6 is rejected.

### Discussion

In study 1, we examined the influence of diversity climate on job satisfaction and inclusion, among a sample of employees working in a highly culturally diverse work environment. We predicted that diversity climate would enhance these outcomes through a mediating effect of trust and openness in workgroup communication. Our results confirm that diversity climate significantly relates to both job satisfaction and inclusion, which is in line with earlier findings (Hofhuis et al. 2012; Otten and Jansen 2014). When testing mediation of workgroup communication, our findings show that only trust appears to be responsible for these positive findings. We conclude that it mediates the relationship with inclusion, as well as finding an indication of a possible mediating effect on the relationship with job satisfaction, although the latter was only significant at the α = .10 level. Considering the relatively small size of the sample in this study, it is not unlikely that significant mediation may be found when testing this hypotheses among a larger group of respondents. Openness, while still positively related to inclusion, does not appear to play a mediating role, which is contrary to what was expected.

As mentioned, the main limitation of this study is its relatively small sample size, which reduced the statistical power in structural equation modeling. Furthermore, this study was conducted in highly diverse work teams, where the presence of a strong diversity climate may be one of the key factors which determine employee communication and team outcomes, which could explain the particularly strong relationships between diversity climate and trust and openness. This study thus confirms that organizations which employ people of many cultural backgrounds within their workgroups would benefit greatly from emphasizing a strong diversity climate. Whether this statement holds in more traditional organizations, characterized by a lesser degree of cultural diversity, will be tested in study 2.

## Study 2

The aims of study 2 were twofold. Firstly, as mentioned above, we aimed to replicate the findings from study 1 in different types of work environments. To improve generalizability of our results, we chose to test the relationships between diversity climate and workgroup communication among employees of a broad range of different organizations within different sectors. The hypothesized effects remain the same.

The second aim of study 2 is to examine the mediating effects of trust and openness on different outcomes. The outcome variables that were included in study 1 were related mostly to the social identity/self-categorization paradigm in diversity research (Van Knippenberg et al. 2004); as employees in diverse teams categorize themselves into cultural subgroups, job satisfaction is reduced, and team members’ sense of inclusion decreases. The results of study 1 show that diversity climate will actually increase these outcomes, and that this is likely to be due to an increase in trust in workgroup communication. In study 2, we will examine whether this finding also holds for *workgroup identification,* another outcome variable which falls along this path. Categorization into cultural subgroups is likely to reduce team cohesion and members’ tendency to psychologically associate with their workgroup. Earlier studies have shown that a strong diversity climate will inhibit these processes and enhance workgroup identification in diverse teams (Hofhuis et al. 2012; Luijters et al. 2008). In study 2, we will examine whether this effect is also mediated by an increase in trust in communication.

### **Hypothesis 7**

The relationship between diversity climate and workgroup identification is mediated by Trust.

Finally, the question remains how diversity climate affects variables that relate to information elaboration, i.e. whether it will enhance the benefits of diversity for workgroups. Our earlier finding that openness in workgroup communication does not mediate the relationship between diversity climate and categorization-related outcomes has prompted us to speculate that this construct may instead be responsible for the relationship between diversity climate and the exchange of knowledge in diverse teams. Indeed, Nakui et al. (2011) have shown that when diversity attitudes in teams are positive, there is a greater chance of information sharing and idea generation among culturally diverse team members. Furthermore, openness in communication has repeatedly been identified as a major predictor of knowledge sharing intentions (Mueller 2014; Van Den Hooff and De Ridder 2004). Based on these findings, we hypothesize that, under the influence of diversity climate, the possibility for open communication enhances the team’s ability to voice divergent viewpoints, ultimately increasing knowledge sharing between employees.

### **Hypothesis 8**

The relationship between diversity climate and knowledge sharing is mediated by openness.

### Methods

#### Procedure and sample

Data for this study were gathered using a digital survey, distributed among a sample of employees working in organizations in the Netherlands. As part of a course assignment, a group of students at the University of Amsterdam was instructed to invite respondents from their personal network. Such student-recruited samples appear to be demographically similar to non-student recruited samples, and this method is not reported to influence validity of the data (Wheeler et al. 2014). The respondents were required to be 18 years or older, and to currently work within an organization for a minimum of 20 h per week. In total, 364 respondents were approached through an e-mail with a link to the digital survey. Respondents were asked to complete the survey within 10 working days. No compensation was given.

The final sample used in this study consisted of 246 respondents who fully completed the questionnaire (response rate = 68 %). Mean age was 37.3 years (SD = 12.2; range: 19–64), 46.7 % was male, 74.8 % had received higher education (defined as a bachelor’s degree or above). Our sample consisted of a cross-section of organizations in the Netherlands, in many different sectors including service industry (22.8 %), public administration (17.1 %), health/social care (9.8 %), communication and media (6.1 %), science and education (6.1 %), trade (5.7 %), finance (5.7 %), manufacturing (4.5 %) and others (22.2 %). Furthermore, 4.9 % (*n* = 12) of the sample belonged to a non-Western minority group, as defined by the country of birth of their parents (in line with the definition of the Netherlands’ Central Bureau of Statistics; CBS 2016), including Turkish, Indonesian, Moroccan, Surinamese, Bosnian, Iraqi, and Pakistani cultural heritage. Unfortunately, the number of minority members in the sample was too small to examine the influence of cultural background on the proposed relationships in our model. The exact cultural composition of the respondents’ teams or organizations is unknown. However, existing data (CBS 2016) show that approximately 11 % of the Dutch workforce consists of non-Western minority employees, so we are assuming that this also holds, on average, for our respondents’ work contexts.

#### Measures

The questionnaire was distributed in Dutch, the main working language of the respondents. All formulations and examples provided hereafter were translated by the authors. All scales described below were measured using a Likert scale, ranging from 1 (*Strongly disagree*) to 5 (*Strongly agree*).

*Diversity Climate* was measured using the original Dutch formulations of four items that were used by Hofhuis et al. (2012; see also study 1). These items included statements such as ‘In this organization there is room to work according to one’s own culture’ and ‘Within this organization we appreciate different cultural backgrounds’. The scale was sufficiently reliable (*α* = .65)

*Trust* and *Openness* in communication were measured using items by Smidts, et al. (2001) from a Dutch-language instrument intended to measure components of an organizational communication climate. Only the subscales for Trust (three items, e.g. ‘When my colleagues tell me something, I can be sure that they are telling the truth’) and openness (three items, e.g. ‘my colleagues are open to hearing my suggestions’) were used. The scales were sufficiently reliable (*α* = .77; .69 respectively).

*Workgroup Identification* was measured using three items translated from Allen and Meyer (1990), including statements such as ‘I am proud to work within this team’ and ‘The problems of the team feel like my own problems’. The scale was reliable (*α* = .81).

*Knowledge Sharing* was measured using five items adapted from Van der Rijt (2007), including statements such as ‘I enjoy sharing my knowledge and experience with my colleagues’. The scale was reliable (*α* = .79).

#### Measurement model

As the constructs in this study are theoretically linked, we performed a confirmatory factor analysis to test the discriminant validity of the scales for diversity climate, trust, openness, workgroup identification and knowledge sharing. First, we tested a one-factor model, which resulted in a poor fit (*χ*^*2*^(122) = 245.112; *p* < .001; CFI = .632; RMSEA = .091) The intended measurement model, which included the five latent variables, provided a good fit (*χ*^*2*^(122) = 209.796; *p* < .001; CFI = .961; RMSEA = .043). However, based on modification indices, we chose to covary the error terms of two of the items measuring diversity climate, as well as those of two items measuring knowledge sharing. This provided a significantly better fitting model (*χ*^*2*^(120) = 143.836; *p* = .068; CFI = .983; RMSEA = .028; *Δχ*^*2*^(2) = 65.96; *p* < .001), which was subsequently used for testing our hypotheses. Table 2 provides descriptive statistics and intercorrelations of all variables in the model.

**Table 2:** Descriptive statistics and correlations of variables in study 2 (n = 246)

Variable	Descriptives		r					
	α	M	SD	(1)	(2)	(3)	(4)	(5)
Diversity climate (1)	.65	3.49	.54	–	.16**	.25***	.14*	.10*
Trust (2)	.77	4.02	.55		–	.36***	.49***	.21***
Openness (3)	.69	3.88	.52			–	.35***	.25***
Workgroup identification (4)	.81	3.71	.61				–	.34***
Knowledge sharing (5)	.79	3.60	.50					–

* p < .05; ** p < .01; *** p < .001

### Results

#### Structural model

**Fig. 2:**
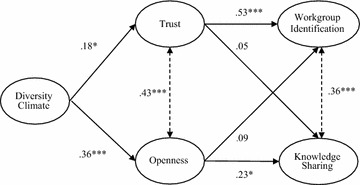
Structural model of effects of diversity climate on workgroup identification and knowledge sharing, mediated by trust and openness (n = 246). *Note* A *double arrow* with a *dotted line* signifies a correlation between error terms of the latent variables; all reported coefficients are standardized; *χ* ^*2*^(125) = 173.680; *p* = .003; CFI = .966; RMSEA = .040; *p < .05; **p < .01; ***p < .001

A structural equation model was constructed based on the hypotheses outlined above, including *Diversity Climate* as an independent variable, *Workgroup Identification* and *Knowledge Sharing* as dependent variables, and *Trust* and *Openness* as mediators (see Fig. 2). In study 1, it was decided to also include error correlations between trust and openness, as well as between the outcome variables, since these constructs are theoretically related to each other. To be consistent, the same was done here. The final model, including error correlations, resulted in a good fit (*χ*^*2*^(125) = 173.680; *p* = .003; CFI = .966; RMSEA = .040; Fig. 2).

#### Direct effects

The standardized estimated regression weights in Fig. 2 show that diversity climate positively predicts both trust (b* = .18; SE = .08, *p* = .024) and openness (b* = .36; SE = .05; *p* < .001) in workgroup communication, thereby confirming findings from study 1. Furthermore, trust is positively related to workgroup identification (b* = .53; SE = .09, *p* < .001), but not related to knowledge sharing (b* = .05; SE = .04; *p* = .122). Openness is positively related to knowledge sharing (b* = 23; SE = .11, *p* = .016), but not to workgroup identification (b* = .09; SE = .07; *p* = .091). Trust and openness display a positive correlation (*r* = .43, *p* < .001), as well as workgroup identification and knowledge sharing (*r* = .36, *p* < .001).

#### Mediations

Hypotheses 7 and 8 predict that the positive effects of diversity climate on workgroup identification and knowledge sharing are mediated through, respectively, trust and openness. Mediations were tested using bootstrapping (5000 iterations). Firstly, a significant total effect was found of diversity climate on workgroup identification (b* = .18; SE = .05; *p* < .001), as well as a significant indirect effect through trust (b* = .13; SE = .05; *p* = .009), thereby confirming hypothesis 7.

Our analyses also reveal a significant total effect of diversity climate on knowledge sharing (b* = .13; SE = .05; *p* < .001), as well as a significant indirect effect through openness (b* = .10; SE = .06; *p* = .005), thereby confirming hypothesis 8.

### Discussion

The aims of study 2 were twofold. Firstly, we aimed to replicate our earlier finding that diversity climate is positively related to both trust and openness in communication, among more generalized sample of employees. Our results provide successful replication. Although the relationships are not as strong as in study 1, which was conducted in a highly culturally diverse work environment, diversity climate also seems to significantly relate to both communication constructs in more normalized work environments across a broad range of organizations and sectors.

Secondly, we aimed to examine the influence of diversity climate on two other outcome variables, namely workgroup identification and knowledge sharing, and test the possible mediating effect of trust and openness in these relationships. As predicted, diversity climate displays a positive effect on workgroup identification. This is in line with findings from study 1, as well as with existing literature (Hofhuis et al. 2012; Singh et al. 2013), which shows that diversity climate reduces categorization into subgroups and may overcome perceived differences among colleagues to enhance cohesion. Moreover, our results show that this effect is mediated through trust, as predicted.

Furthermore, we examined whether diversity climate enhances knowledge sharing in workgroups, a construct which relates to the information elaboration which is often cited as a positive outcome of diversity (Van Knippenberg et al. 2004). As predicted, we found a positive relationship, which was also shown to be mediated through openness.

## General discussion

### Summary of findings

Diversity climate, defined as an organizational climate characterized by openness and/or appreciation towards cultural differences, has emerged as one of the most promising factors determining the success of diversity management in (multicultural) organizations. Earlier studies have provided evidence that promoting a strong diversity climate may be an effective way to minimize negative outcomes, while still retaining the possibility of gaining positive outcomes of cultural diversity (Groggins and Ryan 2013; Hofhuis et al. 2015). To date, however, it remains unclear which processes may be responsible for these effects. Recent reviews of the literature on diversity climate specifically call for a more thorough investigation of possible mediators of the relationships between diversity climate and workgroup outcomes (Boehm et al. 2014; Dwertmann et al. 2016). The present study answers this call, by testing whether workgroup communication could be such a mediator.

In two studies, we first examined whether perceived diversity climate is related to trust and openness in workgroup communication. The results of study 1 show that diversity climate is indeed strongly related to both communication factors in highly culturally diverse teams. Study 2 replicates this result among a sample of employees working in a broad range of organizations within different sectors, which suggests generalizability of our findings. Furthermore, the respondents in study 2 were employed in a range of teams which were not specifically characterized by a high degree of cultural diversity, but instead represent a cross-section of regular organizations in the Netherlands. Although the relationships seems to be less strong in this context, diversity climate still appears to enhance the degree of trust and openness in workgroup communication to a significant extent, even in teams with a limited degree of cultural diversity.

Furthermore, this paper provides evidence that trust and openness mediate the relationships between diversity climate and diversity outcomes. Specifically, trust seems to be responsible for the positive effects of diversity climate on job satisfaction, inclusion and workgroup identification of employees. Furthermore, study 2 shows that openness in workgroup communication mediates the positive relationship between diversity climate and knowledge sharing.

### Theoretical implications

As stated, our findings are in line with a growing body of research which identifies diversity climate as an important factor in diversity management. A major contribution of the present research is that it also provides evidence for the positive effect of diversity climate on knowledge sharing, which indirectly implies an effect on elaboration and creativity within diverse teams. Although these relationships have been found for positive diversity attitudes and certain personality factors (Homan et al. 2008; Nakui et al. 2011), this paper extends the literature in showing that diversity climate, as a specific organizational variable, displays similar effects.

The evidence for workgroup communication as one of the possible mediators opens up new possibilities for explaining how diversity climate enhances workgroup effectiveness. Although communication has been suggested by several authors as an important factor, empirical studies which link communication processes with cultural diversity are scarce, and are focused mainly on explaining differences between cultural groups in communication content and/or style (Dinsbach et al. 2007; Orbe 1998). Our results show that the interplay between cultural diversity and interpersonal communication in the workplace is a promising avenue of exploration, and may enhance our insight in how members of diverse teams interact.

Furthermore, our studies fit within the broader research on organizational climates, and provide a bridge between diversity climate literature (Dwertmann et al. 2016) and more specific studies on communication climates within organizations (e.g. Smidts et al. 2001; Van Den Hooff and De Ridder 2004). Although the findings reported in this paper relate to a psychological climate construct, as opposed to a more group-level organizational climate construct (Martin et al. 2005), we have shown that the climate towards diversity can be a strong predictor of outcomes, and should be regarded as a key component of diversity management in organizations. As such, it confirms existing claims that climate variables are important in predicting and explaining organizational behavior in general (Martin et al. 2005; Schneider et al. 2013). We also predict that diversity climate may be related to other climate constructs, such as ethical, justice or moral climate (Macklin et al. 2014), but this proposition should be empirically tested before making any definite claims.

Finally, our results shed new light on the relationship between trust and openness in workgroup communication, an area which is currently under debate. Although some scholars argue that both constructs are fundamentally intertwined (Ruppel and Harrington 2000; Tjosvold 1999), we have shown that, although correlated, they are separate communication factors which display divergent effects on different organizational outcomes.

### Limitations and directions for future research

The major limitation of the research presented in this paper is its reliance on individual-level measures to operationalize the group-level construct of diversity climate. As explained in the introduction, the climate measure that was employed in both studies reflects the employees’ perceptions about their own workgroup/organization’s diversity climate, and as such should be seen as a psychological climate variable. Although this approach to assessing diversity climate is very common (see Dwertmann et al. 2016), more research is needed to further confirm the effects of group-level diversity climate on group-level outcomes such as workgroup communication. To advance the stream of research on diversity climate in organizations, we recommend future scholars replicate our findings using aggregated measures of organizational climate, and/or through hierarchical linear modeling of diversity climate in workgroups or organizations using a nested design.

Another major limitation of the studies reported in this paper is that the data are cross-sectional. Although in our mediation analyses, we assume a causal relationship, with diversity climate being responsible for enhancing workgroup communication, it is not unlikely that workgroups which display high trust and openness may over time develop a more strong diversity climate. Future studies could address this issue by including a longitudinal analysis of diversity climate and communication factors, or by manipulating these factors in an experimental design.

Thirdly, our data was gathered using single source self-reports only, which means it is possible that some of the reported relationships are influenced by common method variance (Podsakoff et al. 2012). To address this issue in future research, we recommend replicating our results using more divergent measures of workgroup communication and team outcomes. A study by Van Oortmerssen et al. (2014), which uses a structured observation of trust in interpersonal interaction, is a good example of such a methodology. Also, more detailed examination of the content of interpersonal communication in diverse teams could greatly increase our understanding of the more specific communication patterns which occur in such a setting, and how they are affected by diversity climate. Combining our methodology with a more specific measure of communication content (e.g. Dinsbach et al. 2007), would be a logical next step in this research line.

Finally, the aim of the studies presented above was to assess the main effects of diversity climate on workgroup communication and outcomes. Our findings imply that the predicted effects of diversity climate will occur in both highly diverse (study 1) and less diverse (study 2) work contexts. However, it would be very beneficial to study the interaction between diversity climate and actual team diversity, cultural or otherwise, on outcomes. Many studies (e.g. Kearney and Gebert 2009) view diversity in workgroups as a continuum, where the degree of diversity is dependent on the number and size of different cultural groups represented within the team (cf. Harrison and Klein 2007). Future studies could test whether the effects of this type of diversity are moderated by the diversity climate in these teams. Furthermore, in many organizations, diversity is not seen as a continuum; instead, there is a clear distinction between majority and minority employees. Earlier studies (e.g. Hofhuis et al. 2012) have established that the cultural background of employees, measured as a dichotomy, interacts with perceived diversity climate to predict outcomes. It is not unlikely that communication patterns between cultural groups may also be characterized differently (e.g. Dinsbach et al. 2007; Hofhuis et al. 2012). Further inquiry is needed to understand how these processes may be affected by diversity climate.

### Conclusions

Our results are consistent with existing literature in that diversity climate seems to enhance satisfaction, inclusion and identification in teams (Gonzalez and Denisi 2009; Hofhuis et al. 2012; Luijters et al. 2008) as well as increasing the possibility of reaping some of the benefits of diversity through increased knowledge sharing (De Dreu and West 2001; Nakui et al. 2011). Furthermore, workgroup communication is shown to be an important mediator in these processes. In study 1, trust is shown to mediate the effects of diversity climate on team members’ sense of inclusion. In study 2, trust mediates the relationship between diversity climate and workgroup identification and openness mediates its relationship with knowledge sharing.

Our results confirm that diversity climate plays a key role in the success of diversity management in organizations. By increasing the ability of employees to display their cultural heritage in the workplace, and by promoting the ‘value-in-diversity’-perspective, organizations are likely to be more successful in dealing with cultural differences. Earlier research has provided evidence that a positive attitude towards diversity will lead to better job-related outcomes, more innovation, higher customer satisfaction and more equal recruitment policies (Avery and McKay 2006; Van Knippenberg and Schippers 2007). The present research shows that workgroup communication is a particularly important component of these processes. As such, we recommend that organizations which aim to reap the benefits of diversity should focus their efforts on providing training in intercultural communication and enhancing trust and openness in interpersonal interaction. Furthermore, organizations which initiate an organizational change towards a stronger diversity climate would benefit from paying specific attention to these communication patterns, and monitoring how workgroup communication is affected by their efforts. By increasing diversity climate, both trust and openness should be increased. More trust in diverse workgroups is likely to reduce some of the negative outcomes which are often the result of cultural diversity. More openness in workgroup communication may be one of the ways to unlock the potential positive outcomes, and will ultimately make the organization more productive.

## References

[CR1] Allen NJ, Meyer JP (1990). The measurement and antecedents of affective, continuance and normative commitment to the organization. J Occup Psychol.

[CR2] Arbuckle JL (2012). Amos (version 21.0) [Computer program]. IBM SPSS, Chicago

[CR3] Avery DR, McKay PF (2006). Target practice: an organizational impression management approach to attracting minority and female job applicants. Pers Psychol.

[CR4] Bartels J (2006) Organizational identification and communication: employees’ evaluations of internal communication and its effect on identification at different organizational levels. Doctoral dissertation. University of Twente, Enschede

[CR5] Boehm SA, Dwertmann DJG, Kunze F, Michaelis B, Parks KM, McDonald DP (2014). Expanding insights on the diversity climate-performance link: the role of workgroup discrimination and group size. Hum Resour Manag.

[CR07] Brewer MB, Brown RJ (1998) Intergroup relations. In: Gilbert DT, Fiske ST, Lindzey G (eds) The handbook of social psychology, vols. 1, 2, 4th edn. McGraw-Hill, New York, pp 554–594

[CR01] Brodbeck FC, Greitemeyer T (2000). Effects of individual versus mixed individual and group experience in rule induction on group member learning and group performance. J Exp Soc Psychol.

[CR6] Butler J (1999). Trust expectations, information sharing, climate of trust, and negotiation effectiveness and efficiency. Group Organ Manag.

[CR7] Buttner EH, Lowe KB, Billings-Harris L (2012). An empirical test of diversity climate dimensionality and relative effects on employee of color outcomes. J Bus Ethics.

[CR8] CBS (2016) Centraal Bureau voor Statistiek (Netherlands Central Bureau of Statistics) Statline. Retrieved from www.cbs.nl/statline

[CR9] Choi S (2013). Demographic diversity of managers and employee job satisfaction empirical analysis of the federal case. Rev Public Pers Adm.

[CR10] Chrobot-Mason D, Aramovich NP (2013). The psychological benefits of creating an affirming climate for workplace diversity. Group Org Manag.

[CR02] Collins B, Geutzkow H (1964). A social psychology of group processes for decision-making.

[CR11] Costa AC, Roe RA, Taillieu T (2001). Trust within teams: the relation with performance effectiveness. Eur J Work Organ Psychol.

[CR12] Cox TJ (1993). Cultural diversity in organizations, theory, research and practice.

[CR13] Dahlin KB, Weingart LR, Hinds PJ (2005). Team diversity and information use. Acad Manag J.

[CR14] De Dreu CKW, West MA (2001). Minority dissent and team innovation: the importance of participation in decision making. J Appl Psychol.

[CR15] De Vries RE, Van Den Hooff B, De Ridder JA (2006). Explaining knowledge sharing the role of team communication styles, job satisfaction, and performance beliefs. Commun Res.

[CR16] De Witte H, Bouwen R, De Witte K, De Witte H, Taillieu T (2000). Arbeidsethos en jobonzekerheid: Metingen en gevolgen voor welzijn, tevredenheid en inzet op het werk. Van groep naar gemeenschap.

[CR17] Dinsbach AA, Feij JA, De Vries RE (2007). The role of communication content in an ethnically diverse organization. Int J Intercult Relat.

[CR18] Drach-Zahavy A, Trogan R (2013). Opposites attract or attack? The moderating role of diversity climate in the team diversity–interpersonal aggression relationship. J Occup Health Psychol.

[CR19] Dwertmann DJG, Nishii LH, Van Knippenberg D (2016). Disentangling the fairness & discrimination and synergy perspectives on diversity climate: moving the field forward. J Manag.

[CR20] Edmondson A (1999). Psychological safety and learning behavior in work teams. Adm Sci Q.

[CR21] Eisenberg EM, Witten MG (1987). Reconsidering openness in organizational communication. Acad Manag Rev.

[CR22] Fiske ST, Gilbert DT, Fiske ST, Lindzey G (1998). Stereotyping, prejudice and discrimination. Handbook of social psychology.

[CR23] Frey K, Luethje C (2011). Antecedents and consequences of interaction quality in virtual end-user communities. Creativity Innov Manag.

[CR08] Goldberg CB (2005). Relational demography and similarity-attraction in interview assessments and subsequent offer decisions: are we missing something?. Group & Organ Manage.

[CR24] Gonzalez JA, Denisi AS (2009). Cross-level effects of demography and diversity climate on organizational attachment and firm effectiveness. J Organ Behav.

[CR25] Greenhalgh L, Chapman D (1998). Negotiator relationships: construct measurement, and demonstration of their impact on the process and outcomes of negotiation. Group Decis Negot.

[CR26] Groggins A, Ryan AM (2013). Embracing uniqueness: the underpinnings of a positive climate for diversity. J Occup Organ Psychol.

[CR09] Harrison DA, Klein KJ (2007). What's the difference? Diversity constructs as separation, variety, or disparity in organizations. Acad Manag Rev.

[CR27] Hobman EV, Bordia P, Gallois C (2004). Perceived dissimilarity and work group involvement: the moderating effects of group openness to diversity. Group Organ Manag.

[CR28] Hofhuis J, Van der Zee KI, Otten S (2012). Social identity patterns in culturally diverse organizations: the role of diversity climate. J Appl Soc Psychol.

[CR29] Hofhuis J, Van der Zee KI, Otten S (2014). Comparing antecedents of voluntary job turnover among majority and minority employees. Equal Divers Incl.

[CR30] Hofhuis J, Van der Zee KI, Otten S (2015). Measuring employee perception on the effects of cultural diversity at work: development of the benefits and threats of diversity scale. Qual Quant.

[CR31] Hofhuis J, Van der Zee KI, Otten S (2016). Dealing with differences: the impact of perceived diversity outcomes on selection and assessment of minority employees. Int J Hum Resour Manag.

[CR32] Homan AC, Hollenbeck JR, Humphrey SE, Van Knippenberg D, Ilgen DR, Van Kleef GA (2008). Facing differences with an open mind: openness to experience, salience of intragroup differences, and performance of diverse work groups. Acad Manag J.

[CR33] Hooghe M, Reeskens T, Stolle D, Trappers A (2009). Ethnic diversity and generalized trust in Europe A cross-national multilevel study. Comp Political Stud.

[CR34] Jansen WS, Otten S, Van Der Zee KI, Jans L (2014). Inclusion: conceptualization and measurement. Eur J Soc Psychol.

[CR35] Kearney E, Gebert D (2009). Managing diversity and enhancing team outcomes: the promise of transformational leadership. J Appl Psychol.

[CR36] Kruithof H (2001) Multiculturele Persoonlijkheidseigenschappen en Effectiviteit van Allochtone Medewerkers in de Nederlandse Werkorganisatie [Multicultural personality characteristics and efficiency of non-native employees in the Dutch Working Organization] Master Thesis, University of Groningen, Groningen, the Netherlands

[CR37] Kurtzberg TR, Amabile TM (2000). From guilford to creative synergy: opening the black box of team-level creativity. Creativity Res J.

[CR38] Luijters K, Van der Zee KI, Otten S (2008). Cultural diversity in organizations: enhancing identification by valuing differences. Int J Intercult Relat.

[CR39] Macho S, Ledermann T (2011). Estimating, testing, and comparing specific effects in structural equation models: the phantom model approach. Psychol Methods.

[CR40] Macklin R, Martin A, Mathison K (2014). An integrated model of justice and ethical climates and the influence of cultural diversity. Manag Organ Rev.

[CR41] Madera JM, Dawson M, Neal JA (2013). Hotel managers’ perceived diversity climate and job satisfaction: the mediating effects of role ambiguity and conflict. Int J Hosp Manag.

[CR42] Martin AJ, Jones ES, Callan VJ (2005). The role psychological climate in facilitating employee adjustment during organizational change. Eur J Work Organ Psychol.

[CR43] Mayer RC, Davis JH, Schoorman FD (1995). An integrative model of organizational trust. Acad Manag Rev.

[CR44] McKay PF, Avery DR, Tonidandel S, Morris MA, Hernandez M, Hebl MR (2007). Racial differences in employee retention: are diversity climate perceptions the key?. Pers Psychol.

[CR45] McKay PF, Avery DR, Morris MA (2008). Mean racial-ethnic differences in employee sales performance: the moderating role of diversity climate. Pers Psychol.

[CR46] McKay PF, Avery DR, Liao H, Morris MA (2011). Does diversity climate lead to customer satisfaction? it depends on the service climate and business unit demography. Organ Sci.

[CR47] Milliken FJ, Martins LL (1996). Searching for common threads: understanding the multiple effects of diversity in organizational groups. Acad Manag Rev.

[CR48] Milliken FJ, Bartel CA, Kurtzberg TR, Paulus P, Nijstad B (2003). Diversity and creativity in work groups: A dynamic perspective on the affective and cognitive processes that link diversity and performance. Group creativity.

[CR49] Mueller J (2014). A specific knowledge culture: cultural antecedents for knowledge sharing between project teams. Eur Manag J.

[CR50] Nakui T, Paulus PB, Van der Zee KI (2011). The role of attitudes in reactions toward diversity in workgroups. J Appl Soc Psychol.

[CR03] Nijstad BA, De Dreu CK (2002). Creativity and group innovation. Appl Psychol Int Rev.

[CR51] Orbe M (1998). From the standpoint(s) of traditionally muted groups: explicating a co-cultural communication theoretical model. Commun Theory.

[CR52] Otten S, Jansen WS, Otten S, Van der Zee KI, Brewer MB (2014). Predictors and consequences of exclusion and inclusion at the culturally diverse workplace. Towards inclusive organizations: determinants of successful diversity management at work.

[CR53] Parker CP, Baltes BB, Young SA, Huff JW, Altmann RA, Lacost HA, Roberts JE (2003). Relationships between psychological climate perceptions and work outcomes: a meta-analytic review. J Organ Behav.

[CR54] Patterson MG, West MA, Shackleton VJ, Dawson JF, Lawthom R, Maitlis S (2005). Validating the organizational climate measure: links to managerial practices, productivity and innovation. J Organ Behav.

[CR55] Pelled LH, Eisenhardt KM, Xin KR (1999). Exploring the black box: an analysis of work group diversity, conflict, and performance. Adm Sci Q.

[CR56] Pless N, Maak T (2004). Building an inclusive diversity culture: principles, processes and practice. J Bus Ethics.

[CR57] Podsakoff PM, MacKenzie SB, Podsakoff NP (2012). Source of method bias in social science research and recommendations on how to control it. Annu Rev Psychol.

[CR58] Roberson Q (2006). Disentangling the meanings of diversity and inclusion in organizations. Group Organ Manag.

[CR59] Rogers DP (1987). The development of a measure of perceived communication openness. J Bus Commun.

[CR60] Ruppel C, Harrington S (2000). The relationship of communication, ethical work climate, and trust to commitment and innovation. J Bus Ethics.

[CR61] Sadri G, Tran H (2002). Managing your diverse workforce through improved communication. J Manag Dev.

[CR62] Schachner MK, Noack P, Van de Vijver FJR, Eckstein K (2016) Cultural diversity climate and psychological adjustment at school—equality and inclusion versus cultural pluralism. Child Development. doi:10.1111/cdev.12536 10.1111/cdev.12536 27091829

[CR63] Schermelleh-Engel K, Moosbrugger H, Müller H (2003). Evaluating the fit of structural equation models: tests of significance and descriptive goodness-of-fit measures. Methods Psychol Res.

[CR64] Schneider B, Ehrhart MG, Macey WH (2013). Organizational climate and culture. Annu Rev Psychol.

[CR65] Shore LM, Randel AE, Chung BG, Dean MA, Ehrhart KH, Singh G (2011). Inclusion and diversity in work groups: a review and model for future research. J Manag.

[CR66] Singh B, Winkel DE, Selvarajan T (2013). Managing diversity at work: does psychological safety hold the key to racial differences in employee performance?. J Occup Organ Psychol.

[CR67] Smidts A, Pruyn A, Van Riel C (2001). The impact of employee communication and perceived external prestige on organizational identification. Acad Manag J.

[CR06] Tajfel H, Turner JC (1986) The social identity theory of intergroup behavior. In: Psychology of intergroup relations, The Nelson-Hall series psychology, vol 2. Nelson-Hall, Chicago, pp 7–24

[CR68] Tjosvold D (1999). Bridging east and west to develop new products and trust: interdependence and interaction between a Hong Kong parent and North American subsidiary. Int J Innov Manag.

[CR05] Turner JC (1985). Social categorization and the self-concept: a social-cognitive theory of group behavior. Adv Group Process.

[CR69] Van Den Hooff B, De Ridder JA (2004). Knowledge sharing in context: the influence of organizational commitment, communication climate and CMC use on knowledge sharing. J Knowl Manag.

[CR70] Van der Rijt PGA (2007) Precious knowledge: Virtualness and the willingness to share knowledge in organizational teams. Doctoral dissertation, University of Amsterdam, the Netherlands

[CR71] Van der Zee K, Vos M, Luijters K (2009). Social identity patterns and trust in demographically diverse work teams. Soc Sci Inf.

[CR72] Van Knippenberg D, Schippers MC (2007). Work group diversity. Annu Rev Psychol.

[CR73] Van Knippenberg D, De Dreu CKW, Homan AC (2004). Work group diversity and group performance: an integrative model and research agenda. J Appl Psychol.

[CR74] Van Knippenberg D, Van Ginkel WP, Homan AC (2013). Diversity mindsets and the performance of diverse teams. Organ Behav Hum Decis Process.

[CR75] Van Oortmerssen LA, Van Woerkum CM, Aarts N (2014). The visibility of trust: exploring the connection between trust and interaction in a dutch collaborative governance boardroom. Public Manag Rev.

[CR76] Victor BC, Cullen JB (1988). The organizational bases of ethical work climate. Adm Sci Q.

[CR77] Wanguri DM (1996). Diversity, perceptions of equity, and communicative openness in the workplace. J Bus Commun.

[CR04] West MA (2002). Ideas are ten a penny: it’s team implementation not idea generation that counts. Appl Psychol Int Rev.

[CR78] Wheeler AR, Shanine KK, Leon MR, Whitman MV (2014). Student-recruited samples in organizational research: a review, analysis, and guidelines for future research. J Occup Organ Psychol.

[CR79] Williams K, O’Reilly C (1998). Demography and diversity in organizations: a review of 40 years of research. Res Organ Behav.

